# Effect of ovary induction on bread wheat anther culture: ovary genotype and developmental stage, and candidate gene association

**DOI:** 10.3389/fpls.2015.00402

**Published:** 2015-06-18

**Authors:** Ana M. Castillo, Rosa A. Sánchez-Díaz, María P. Vallés

**Affiliations:** Departamento de Genética y Producción Vegetal, Estación Experimental de Aula Dei, Consejo Superior de Investigaciones Científicas (EEAD-CSIC)Zaragoza, Spain

**Keywords:** wheat, anther culture, ovary co-culture, ovary developmental stage, ovary genotype, fasciclin-like arabinogalactan, *FERONIA*

## Abstract

Ovary pre-conditioned medium and ovary co-culture increased the efficiency of green doubled haploid plant production in bread wheat anther culture. The positive effect of this medium led to a 6- and 11-fold increase in the numbers of embryos and green plants, respectively, having a greater effect on a medium-low responding cultivar. Ovary genotype and developmental stage significantly affected microspore embryogenesis. By the use of Caramba ovaries it was possible to reach a 2-fold increase in the number of embryos and green plants, and to decrease the rate of albinism. Mature ovaries from flowers containing microspores at a late binucleate stage raised the number of embryos and green plants by 25–46% as compared to immature ovaries (excised from flowers with microspores at a mid-late uninucleate stage). The highest numbers of embryos and green plants were produced when using mature Caramba ovaries. Ovaries from Galeón, Tigre, and Kilopondio cultivars successfully induced microspore embryogenesis at the same rate as Caramba ovaries. Moreover, Tigre ovaries raised the percentage of spontaneous chromosome doubling up to 71%. Attempts were made to identify molecular mechanisms associated to the inductive effect of the ovaries on microspore embryogenesis. The genes *TAA1b*, *FLA26*, and *WALI6* associated to wheat microspore embryogenesis, the *CGL1* gene involved in glycan biosynthesis or degradation, and the *FER* gene involved in the ovary signaling process were expressed and/or induced at different rates during ovary culture. The expression pattern of *FLA26* and *FER* could be related to the differences between genotypes and developmental stages in the inductive effect of the ovary. Our results open opportunities for new approaches to increase bread wheat doubled haploid production by anther culture, and to identify the functional components of the ovary inductive effect on microspore embryogenesis.

## Introduction

The production of doubled haploid (DH) plants is a valuable tool for plant breeding, genetics and functional genomics (Forster et al., 2007; Ferrie et al., 2011; Germanà, 2011), as it is a rapid, direct method to obtain homozygous plants. Of all the different methods for bread wheat (*Triticum aestivum* L.) DH production, microspore embryogenesis, through anther or microspore culture, has the greatest potential. However, this technique has major limitations due to the low percentage of microspores that enter the sporophytic pathway, the high percentage of albinism of many genotypes and the low percentage of spontaneous chromosome doubling (for review see Jauhar et al., 2009; Lantos et al., 2013). Therefore, the lack of protocols that can be successfully applied to a broad range of genotypes is still a bottleneck for bread wheat DH production.

Both anther and isolated microspore cultures are based on the swicth of microspores from their normal pollen development toward an embryogenic pathway. However, each method had specific requirements (Soriano et al., 2007). In isolated bread wheat microspore culture, the presence of “nurse factors” in the culture medium can be critical in order to initiate and sustain microspore embryogenesis efficiently (Zheng et al., 2002). Although different flower tissues have been assayed as “nurse factor” donors, namely anthers, ovaries, glumes, and whole florets, ovaries are used most frequently. Ovary co-culture and/or an ovary pre-conditioned medium (in which ovaries have been grown for different time periods, OVCM), had been reported to increase embryogenesis and green plant regeneration in isolated wheat microspore cultures (Mezja et al., 1993; Puolimatka et al., 1996; Hu and Kasha, 1997; Puolimatka and Pauk, 1999; Zheng et al., 2002). Furthermore, the use of OVCM can be essential in order to produce embryos in recalcitrant genotypes (Zheng et al., 2002).

In bread wheat anther culture, early studies suggested that ovary co-culture stimulated regeneration, but it was not a prerequisite for high efficiency (Datta and Wenzel, 1987), as the anther wall could provide the necessary “nurse factors” (Bruins et al., 1996). Therefore, ovary co-culture and/or ovary pre-conditioned media have not traditionally been used. However, Broughton (2008) demonstrated the beneficial effect of ovary co-culture on embryo production in anther cultures of different Australian bread wheat cultivars. Ovary co-culture has occasionally been used in anther cultures by Soriano et al. (2007); Chauhan and Khurana (2011) and Tadesse et al. (2013). In durum wheat, ovary co-culture also enhanced the number of embryo and calli in anther culture, but had no effect on green plant regeneration (J'Aitil et al., 1999).

In isolated microspore cultures, it has been shown that ovaries at different developmental stages have a promoting effect on embryogenesis (Zheng et al., 2002). However, in most studies immature ovaries excised from the flowers used for microspore culture (with microspores at a mid-late uninucleate stage) are used for co-culture or OVCM. In anther cultures, immature and mature ovaries (from flowers containing microspores at a late bininucleate stage) have been used by Broughton (2008) and Soriano et al. (2007), respectively. In both anther and isolated microspore culture, ovaries of the same genotype as the anthers are normally used (Hu and Kasha, 1997; Kunz et al., 2000; Broughton, 2008). Furthermore, Zheng et al. (2002) reported that a wide range of genotypes promoted microspore embryogenesis in isolated microspore culture. However, there are no reports of assays to determine the effect of ovary genotypes and their developmental stage for ovary co-culture or OVCM on wheat anther culture.

Although different authors have addressed the identification of the functional compounds released by the ovaries, their nature and the basis of the inductive effect on microspore embryogenesis, still remain unknown (El-Tantawy et al., 2013). Different promoting compounds have been proposed as candidate “nurse factors,” including the auxin PAA or its analogs, organic substances that buffer the medium, or compounds that metabolize the excess of nitrogen or detoxify the culture medium (Ziauddin et al., 1992; Hu and Kasha, 1997; Puolimatka and Pauk, 1999). Great attention has been paid to the arabinogalactan proteins (AGPs), as they were identified in microspore culture medium of barley and maize and promoted zygotic and microspore embryogenesis in maize (Paire et al., 2003; Borderies et al., 2004). Correspondingly, specific *AGP* genes were expressed in anther and isolated microspore during culture, such as *ECA1* in barley (Vrinten et al., 1999), *TaAGP31-like* in wheat (Sánchez-Díaz et al., 2013), *AGP15, AGP23, AGP2*, and *BnAGP Sta 39-4* in rapeseed (Joosen et al., 2007; Malik et al., 2007; Tsuwamoto et al., 2007; El-Tantawy et al., 2013). Moreover, the incorporation of AGP sources, Larcoll and arabic gum in the culture medium with or without ovary co-culture enhanced embryo production in wheat (Letarte et al., 2006). However, other proteins were also identified in the maize microspore culture medium, including one cell-wall invertase, two thaumatins, one 1-3 beta-glucanase, two chitinases and some oligosaccharides (Borderies et al., 2004). Furthermore, it is known that more than 25,000 genes were expressed in ovaries during at least one stage of development and 61.5% occurred in all stages, including genes associated to zygotic and microspore embryo development such as BABY BOOM (BBM) (Boutilier et al., 2002), and genes associated with ovary-pollen interactions, such as the defensin-like LURE or cysteine-rich proteins (Tran et al., 2013).

The objective of this work was to evaluate the effect of ovary genotype and ovary developmental stage on the efficiency of bread wheat anther culture when an ovary pre-conditioned medium and ovary co-culture were used. Furthermore, attempts were made to identify candidate genes associated with the ovary induction effect.

## Materials and methods

### Material and growing conditions of the donor plants

The spring cultivars of bread wheat “Pavon” and “Caramba” were used as donor plants for anther culture and ovary co-culture. “Pavon” has a high microspore embryogenesis response, whereas “Caramba” has a medium-low response. The agronomically important cultivars Galeón (spring), Tigre (alternative), and Kilopondio (alternative) were also used for ovary co-culture. Donor plants were grown as described by Soriano et al. (2007).

### Preparation of ovary pre-conditioned medium and ovary co-culture (OVPCM)

Ovaries were excised from flowers that contained microspores at a late binucleate stage of development, unless stated otherwise. Ovaries from the same spike were cultured in MS3M medium [MS medium modified by Hu and Kasha (1997), containing 62 g/l maltose, 1 mg/l 2,4-dichlorophenoxyacetic acid (2,4-D), 1 mg/l benzyladenine (BA)], supplemented with 200 g/l Ficoll type 400 (MS3MF200) (Soriano et al., 2008). Cultures were kept in the dark at 25°C for 5 days before anther culture. The ovaries (eight ovaries per 1.5 ml) were kept in MS3MF200 medium during anther culture (co-culture, OVPCM).

### Anther culture

The microspore developmental stage was determined by DAPI (4′, 6′-diamidino-2-phenylindole) staining (Vergne et al., 1987). Anthers containing the majority of microspores at the mid- to late-uninucleate stage were pre-treated for 5 days in 127.5 g l^−1^ mannitol, 5.9 g l^−1^ CaCl_2_ plus macronutrients from FHG medium (Hunter, 1987) solidified with 8 g l^−1^ Sea Plaque agarose (Soriano et al., 2008).

After pre-treatment, 30 anthers were inoculated in 1.5 ml of OVPCM medium, unless stated otherwise.

#### Experiment 1. effect of OVPCM medium on anther culture response

After mannitol stress treatment, anthers from the same spike were randomly distributed on 3 cm Petri dishes containing 1.5 ml of control medium (MS3MF200) or 1.5 ml of OVPCM. Pavon and Caramba ovaries were used in this experiment for OVPCM preparation.

#### Experiment 2. effect of the ovary genotype and ovary developmental stage used in OVPCM on anther culture response

Pavon and Caramba ovaries from spikes containing microspores at a late binucleate stage (mature ovaries) and microspores at a mid-late uninucleate stage (immature ovaries) were cultured in MS3MF200 for OVPCM preparation. Mannitol pre-treated anthers from Pavon and Caramba were randomly distributed in OVPCM medium with mature or young ovaries from Pavon and Caramba.

#### Experiment 3. candidate ovary genotype for OVPCM

OVPCM was prepared with mature ovaries from the Caramba, Galeón, Tigre, and Kilopondio cultivars. Anthers from Pavon and Caramba were used in this experiment.

Cultures from all experiments were kept in the dark at 25°C. After 10–12 days, plates were replenished with 1.5 ml of the MS3MF400 (MS3M supplemented with 400 g/l Ficoll 400). After 30 days, embryos were transferred to J25-8 medium (Jensen, 1977) for regeneration. Embryos were kept in the dark at 25°C for 2 days and then transferred to the light. Ploidy analysis was performed with a PAS cytometer (Partec) as described by Soriano et al. (2007).

### Statistical analysis

All the experiments were performed with at least two different batches of plants. Each experiment consisted of 10–12 replicates of 30–35 anthers per treatment and genotype. Within replicates, pre-treated anthers from the same spike were randomly distributed between treatments. The following variables were recorded: number of embryos, number of green and albino plants and number of DH plants, all per 100 anthers. Percentage of regeneration (number of regenerated plants per 100 embryos), percentage of green plants (number of green plants per 100 total plants) and percentage of spontaneous chromosome doubling (number of doubled haploids per 100 analyzed plants) were calculated. All experiments were established in a completely randomized design. Statistical analysis was performed using SAS software (SAS Institute Inc., Cary, NC, and Version 9.1). Normality and homogeneity of variance were tested using Kolmogorov-Smirnoff and Levene's tests, respectively. Data were transformed using the square root (x + 0.5) to meet parametric assumptions, except percentage of regeneration and percentage of green plants that did not need transformation. The GLM (Generalized Linear Model) procedure was used to conduct the ANOVA for all variables except for chromosome doubling percentage, which was analyzed using the FREQ procedure to do the Chi Square test. The Duncan method (α ≤ 0.05) was used for the Mean separation.

### Selection of candidate genes, RNA isolation, and quantitative RT-PCR

Candidate genes were selected from the genes associated to the initial phases of microspore embryogenesis in wheat (Sánchez-Díaz et al., 2013) that were expressed in ovaries cultured in OVPCM by semi-quantitative RT-PCR analysis (data not shown): Ta.5024.1 (*WHEAT ALUMINIUM INDUCED-6*, *WALI6*), Ta.9528.1 (a fatty acyl CoA-reductase, *Ta.ANTHER-SPECIFIC1*, *TAA1b*), and Ta.1839.1 (*FASCICLIN-26*, *FLA26*). Two genes also expressed in ovaries cultured in OVPCM were included in the analysis: Ta.13696.1 (*COMPLEX GLYCAN LESS 1*, *CGL1)*, a gene previously reported to be associated with AGPs or their derivate, and Ta.14561.2 (*FERONIA*, *FER*), a receptor-like kinase gene associated to ovary signaling processes. The consensus sequence of wheat genes based on HarvEST:Wheat v. 1.59 was used for designing specific primer pairs by PRIMEREXPRESS software (Applied Biosystems) (Table S1).

For RNA isolation, mature and immature ovaries from Pavon and Caramba at 0, 5, 10, and 15 days of culture in MS3MF200 medium (0dC, 5dC, 10dC, and 15dC) were frozen in liquid nitrogen. Mature ovaries from Galeón, Tigre and Kilopondio were frozen at 0dC, 5dC, 10dC, 15dC, and 20dC. Total RNA was isolated from frozen samples using TRIzol Reagent (Gibco BRL), and passed through RNeasy columns (Qiagen) for further clean up, following the manufacturer's instructions in both cases. Double-stranded cDNA was synthesized from the poly(A)+ mRNA present in the isolated total RNA (10.0 μg total RNA) using the M-MLV Reverse Transcriptase kit from Promega. Real-time qPCR reactions were performed with the Fast Maxima SYBR Green Master Mix (Applied Biosystems) and Rox (Rox Reference Dye, Invitrogen). qPCRs reactions for primer-dimer formation and primer efficiency were also performed (data not shown). The reaction conditions were optimized to 95°C for 10 min, followed by 40 cycles of 95°C for 15 s, 60°C for 1 min and a dissociation curve from 65 to 95°C was plotted after each run in the PCR7500 Fast Real-Time PCR System (Applied Biosystems), using gene Ta.27771 as reference (Paolacci et al., 2009). Data from three biological and two technical replicates were analyzed, using the Livak calculation method (Livak and Schmittgen, 2001).

## Results

### Anther culture efficiency

#### Experiment 1. effect of OVPCM medium on anther culture response

Anther culture response was studied in control (MS3MF200 medium) and OVPCM (MS3MF200 ovary pre-conditioned medium and ovary co-culture) cultures, using anthers of the high-responding cultivar Pavon and the medium-low responding cultivar Caramba (Table 1). As expected, Pavon rendered a significantly higher number of embryos, green and albino plants than Caramba (3–5 times higher). Regeneration percentage from Pavon was also higher than from Caramba but both cultivars produced a similar percentage of green plants. Significant differences between control and OVPCM media were observed for all the variables studied. OVPCM gave rise to a 6-fold and 11-fold increase in the number of embryos and green plants, respectively. However, the percentages of plant regeneration and green plants were reduced by 40 and 30%, respectively in OVPCM medium.

**Table 1:** **Effect of ovary pre-conditioned medium and ovary co-culture (OVPCM) on anther culture of bread wheat cultivars Pavon and Caramba**.

**Source of variation *p*-value^2^**	**Embryos**	**Green Pl**	**Albino Pl**	**Reg (%)**	**Green Pl (%)**
Genotype (G)	<0.001	<0.001	<0.001	<0.001	0.103
Culture medium (T)	<0.001	<0.001	0.045	<0.001	<0.001
G x T	0.097	0.001	0.176	0.021	0.065
Batch	0.014	0.017	0.024	0.124	0.051
R^2^	0.80	0.84	0.34	0.63	0.50
**ANTHER GENOTYPE**					
Pavon	309.97a^*^	148.53a	18.67a	48.79a	77.65a
Caramba	92.18b	31.54b	5.00b	29.62b	85.94a
**OVPCM**					
Control	57.64 b^*^	15.94b	8.42b	48.82a	89.77a
OVPCM	369.08a	177.21a	16.60a	31.42b	64.22b

*p-values of the analysis of variance for the numbers of embryos, green plants, albino plants, all of them referred to 100 anthers, and percentages of regeneration and green plants*.

* *Values followed by the same letter within cultivar and treatment are not significantly different (P < 0.05)*.

**Figure 1:**
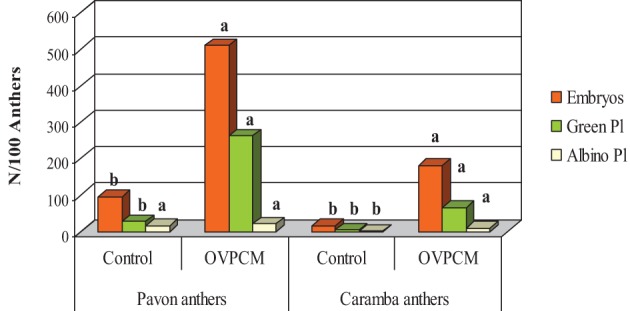
**Pavon and Caramba anther culture response in control medium (nor ovary co-culture neither ovary preconditioned medium) and ovary preconditioned medium with ovary co-culture (OVPCM)**.

A significant anther genotype x culture medium interaction was observed for the number of green plants and the regeneration percentage, OVPCM showing a larger positive effect in the lower-responding cultivar Caramba (Table 1 and Figure 1). In Pavon, 262 green plants were obtained with OVPCM and 27.2 in the control medium, representing a 9.6-fold increase. However, a 16.7-fold increase was observed with OVPCM in Caramba (from 3.8 to 63.8). The regeneration percentage was also enhanced, a 1.3 and a 4-fold increase being observed in Pavon and Caramba with OVPCM, respectively.

#### Experiment 2. effect of the ovary genotype and ovary developmental stage used for OVPCM on anther culture response

**Figure 2:**
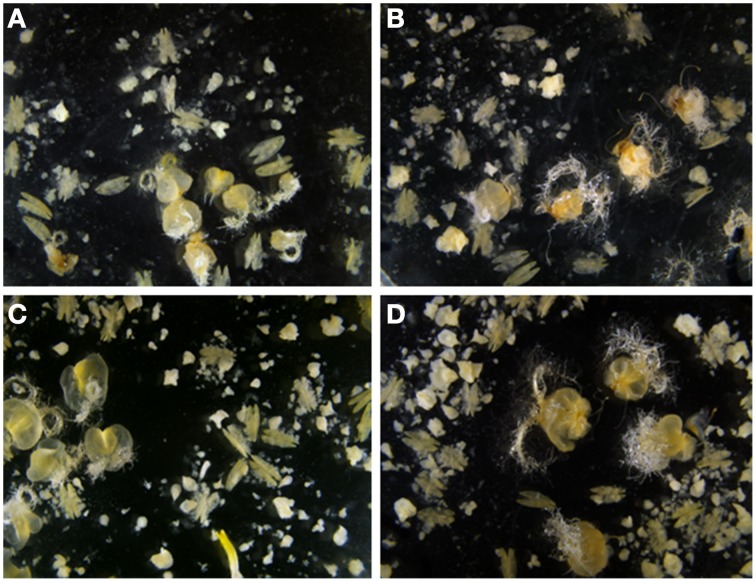
**Effect of the ovary developmental stage and ovary genotype used for OVPCM on Pavon anther cultures. (A)** OVPCM with Pavon immature ovaries. **(B)** OVPCM with Pavon mature ovaries. **(C)** OVPCM with Caramba immature ovaries. **(D)** OVPCM with Caramba mature ovaries.

Immature and mature ovaries corresponding to mid-late uninucleate and late binucleate stages from Pavon and Caramba were used for OVPCM preparation. ANOVA analysis showed that the numbers of embryos and green plants were affected not only by the anther genotype, but also by the ovary genotype and ovary developmental stage (Table 2). Once more, Pavon anther cultures produced a 6-times higher number of green plants than Caramba. When the ovary genotype effect was studied, Caramba ovaries rendered twice as many embryos and green plants as Pavon (Figure 2). A greater percentage of green plants was also obtained with Caramba ovaries. Finally, significant differences were also found between ovary developmental stages (Table 2 and Figure 2). Mature ovaries significantly raised the number of embryos (25%), the number of green plants (46%), and percentage of green plants (17%), in comparison to immature ovaries.

**Table 2:** **Effect of ovary genotype and ovary developmental stage used for OVPCM on anther culture of Pavon and Caramba cultivars of bread wheat**.

**Source of variation *p*-value^2^**	**Embryos**	**Green Pl**	**Albino Pl**	**Reg (%)**	**Green Pl (%)**
An Gen(1)	<0.001	<0.001	0.055	<0.004	<0.001
Ov Gen(2)	<0.001	<0.001	0.008	0.103	<0.001
An Gen x Ov Gen	0.042	0.001	0.018	0.030	0.017
Ov Stage (3)	0.048	0.011	0.513	0.334	0.041
An Gen x Ov Stage	0.476	0.118	0.887	0.530	0.187
Ov Gen x Ov Stage	0.069	0.056	0.272	0.225	0.242
1 × 2 × 3	0.227	0.210	0.781	0.314	0.284
Batch	<0.001	<0.001	<0.001	0.129	<0.001
R^2^	0.60	0.68	0.26	0.24	0.48
**ANTHER GENOTYPE**					
Pavon	242.32 a^*^	143.02a	52.84a	79.78a	63.39a
Caramba	75.72b	24.03b	27.63a	69.82b	37.15b
**OVARY GENOTYPE**					
Pavon	112.87 b^*^	58.78b	40.01b	78.70a	47.58b
Caramba	219.05a	125.14a	43.35a	71.75a	55.94a
**OVARY STAGE**					
Immature	148.63 b^*^	72.42b	40.82a	73.77a	47.54b
Mature	186.08a	106.21a	42.26a	76.76a	55.52a

*p-values of the analysis of variance for the numbers of embryos, green plants, albino plants, all of them referred to 100 anthers, and percentages of regeneration and green plants*.

* *Values followed by the same letter within anther genotype, ovary genotype, and ovary stage are not significantly different (P < 0.05)*.

**Figure 3:**
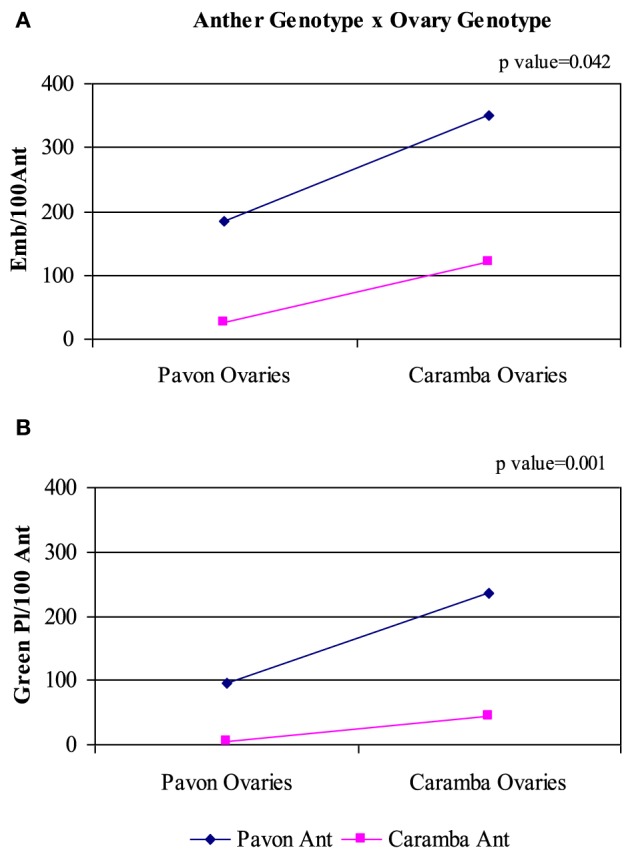
**Analysis of the interaction anther genotype x ovary genotype in anther culture of bread wheat cultivars Pavon and Caramba. (A)** Number of embryos per 100 anthers. **(B)** Number of green plants per 100 anthers.

Caramba ovaries enhanced the anther culture response of both cultivars, but a significant anther genotype x ovary genotype interaction was detected for all the variables studied (Table 2). In the low-responding cultivar Caramba, the number of embryos and green plants increased 5 and 10 times, with Caramba ovaries (Figures 3A,B). However, in the high-responding cultivar Pavon, Caramba ovaries produced only a 2 and 2.5 times higher percentage of embryos and green plants.

No significant ovary genotype x ovary developmental stage interaction was observed for any of the variables; however this interaction was close to the significance level for number of embryos and green plants with *p*-values of 0.07 and 0.06, respectively (Table 2). The positive effect of OVPCM with mature ovaries from Pavon as compared to immature ovaries was higher than that observed between Caramba ovaries. Thus, OVPCM with mature ovaries from Pavon produced a 50% higher number of embryos than immature ovaries (Figures 2A,B), whereas a 15% higher number of embryos was produced with mature ovaries from Caramba (Figures 2C,D). A similar trend was observed for the number of green plants (data not shown). However, we should point out that the highest number of embryos and green plants was obtained with mature ovaries from Caramba and the lowest with immature ovaries from Pavon (Figures 2A,D).

#### Experiment 3. candidate ovary genotype for OVPCM

**Figure 4:**
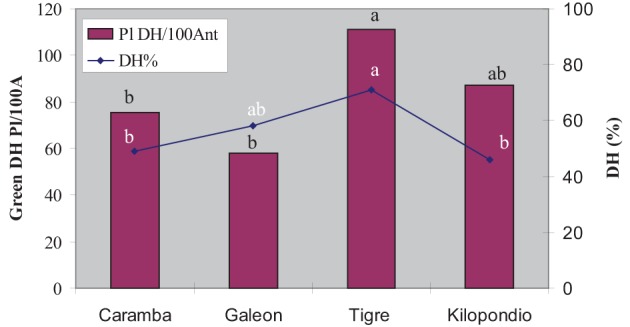
**Number of DH plants per 100 anthers and percentage of spontaneous chromosome doubling produced from wheat anther culture with different ovaries genotypes used for OVPCM**.

Our aim was to find out whether ovaries from other wheat cultivars could further improve the number of green plants in anther culture. Thus, the agronomically important cultivars Galeón, Tigre, and Kilopondio were assayed as donors of mature ovaries for OVPCM and were compared with mature ovaries from Caramba, which had shown the highest inductive effect. Nor were statistically significant differences observed between cultivars for number of embryos and green plants, or for the regeneration and green plant percentages (Table 3). However, differences in the final number of green DH plants were observed between ovary genotypes since they induced distinct rates of spontaneous chromosome doubling (Figure 4). The highest percentage of DH plants was obtained with ovaries from Tigre (71%), followed by Galeón with 58%, whereas Caramba and Kilopondio produced similar percentages of chromosome doubling (45–50%). Accordingly, Tigre ovaries produced the highest number of DH plants, with 111 DH plants/100 anthers. Although Galeón ovaries rendered the highest number of embryos and a high percentage of spontaneous chromosome doubling, this cultivar produced the lowest number of DH plants (58 DH plant/100 anthers), due to its low regeneration and green plant percentage.

**Table 3:** **Effect of ovary genotype used for OVPCM on anther culture of Pavon and Caramba cultivars of bread wheat**.

**Source of variation *p*-value^2^**	**Embryos**	**Green Pl**	**Albino Pl**	**Reg (%)**	**Green Pl (%)**
An Gen	<0.001	<0.001	0.661	<0.001	<0.001
Ov Gen	0.776	0.943	0.277	0.715	0.378
An Gen x Ov Gen	0.714	0.990	0.439	0.296	0.613
Batch	0.701	0.018	0.545	<0.001	0.061
R^2^	0.29	0.45	0.09	0.43	0.45
**ANTHER GENOTYPE**					
Pavon	300.16a^*^	166.79*a*	71.91*a*	80.99*a*	65.86*a*
Caramba	148.39*b*	38.23*b*	67.17*a*	71.07*b*	36.88*b*
**OVARY GENOTYPE**					
Caramba	240.34a^*^	130.32*a*	69.78*a*	79.74*a*	54.90*a*
Galeon	298.06*a*	135.12*a*	84.86*a*	77.80*a*	54.92*a*
Tigre	242.99*a*	123.53*a*	64.04*a*	76.06*a*	60.11*a*
Kilopondio	235.22*a*	121.54*a*	63.20*a*	78.35*a*	58.31*a*

*p-values of the analysis of variance for the numbers of embryos, green plants, albino plants, all of them referred to 100 anthers, and percentages of regeneration and green plants*.

* *Values followed by the same letter within anther genotype and ovary genotype are not significantly different (P < 0.05)*.

### Gene expression analysis

**Figure 5:**
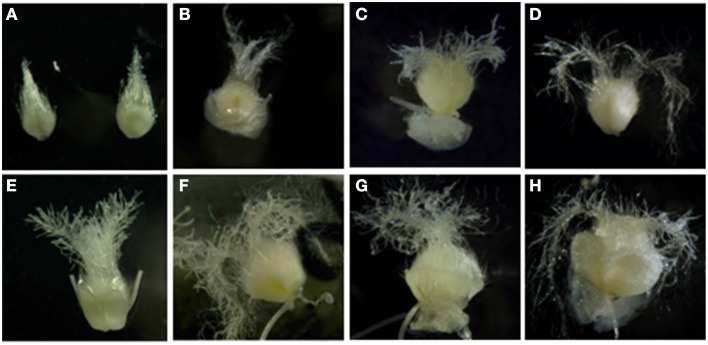
**Immature (A–D) and mature (E–H) ovaries from Caramba in OVPCM at 0 (A,E), 5 (B,F), 10 (C,G) and 15 days of culture (D,H)**.

In order to identify the molecular mechanisms associated to the ovaries' inductive effect on microspore embryogenesis, genes associated to wheat microspore embryogenesis, glycan biosynthesis or degradation, as well as to the ovary signaling process, were considered. Candidate genes were selected based on their expression in Caramba mature ovaries cultured in OVPCM by semi-quantitative RT-PCR analysis (data not shown). The expression pattern of the candidate genes (*WALI6*, *FLA26*, *TAA1b*, *CGL1*, and *FER*) was characterized by quantitative RT-PCR in immature and mature ovaries from Pavon and Caramba at 0, 5, 10, and 15 days of culture (0dC, 5dC, 10dC, and 15dC). At 0dC, mature ovaries were bigger with a more developed stigma than immature ovaries (Figures 5A,E). Both mature and immature ovaries from Caramba enlarged during culture and their stigmatic branches spread, showing a similar morphology to the pollen reception stage at 10dC and 5dC in immature and mature ovaries, respectively (Figures 5B–D,F–H).

**Figure 6:**
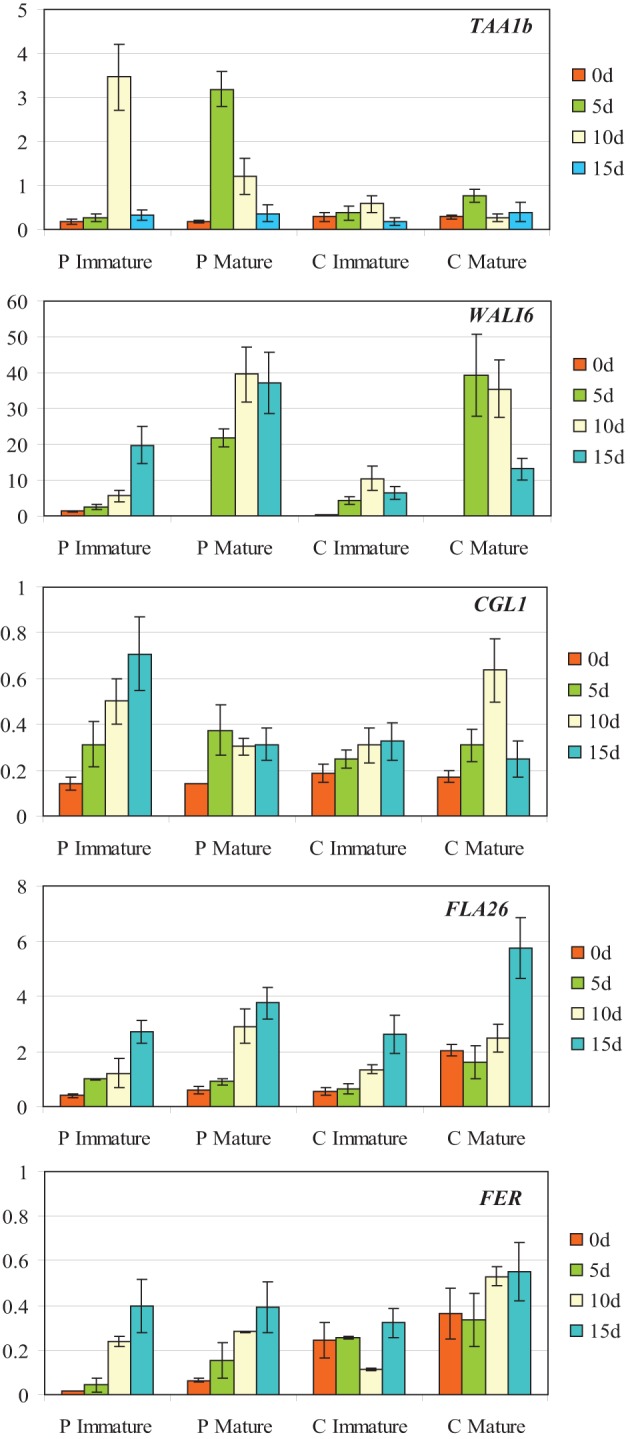
**Expression analysis of *TAA1B*, *WALI6*, *CGL1, FLA26*, and *FERONIA* by qRT-PCR at 0, 5, 10, and 15 days of culture (0dc, 5dc,10dc,15dc, and 20dc) in immature and mature ovaries from bread wheat cultivars Pavon (P) and Caramba (C)**. The *y* axis indicates the gene mRNA level relative to Ta27771 used as control.

Differences in gene expression patterns were observed between ovary genotypes and ovary developmental stages (Figure 6). The gene *TAA1b* that encoded for a fatty acyl CoA-reductase, showed the greatest difference between genotypes, as the gene was only strongly induced in Pavon immature and mature ovaries at 10dC and 5dC, respectively. *WALI6*, a cysteine-rich serine protease inhibitor, showed the highest expression level during culture and the greatest differences between ovary developmental stages. This gene was induced after 5dC in Pavon and Caramba mature ovaries, and reached the highest level of expression at 5dC in Caramba and at 10dC in Pavon. *CGL1*, that encodes the first enzyme in the complex glycan biosynthesis pathway, showed a different expression pattern depending on ovary genotype and developmental stage. In immature ovaries, expression increased during culture in both genotypes, reaching higher levels in Pavon. In mature ovaries, the highest level was observed at 10dC in Caramba.

The two following genes, the fasciclin-like arabinogalactan gene *FLA26* and the receptor-like kinase *FER*, increased their expression during culture, in both genotypes and in both ovary developmental stages (Figure 6). However, it is noteworthy that mature Caramba ovaries, which produced the highest inductive effect on microspore embryogenesis, showed the highest level of expression at the beginning of culture (0dC) and at the latest stage (15dC).

**Figure 7:**
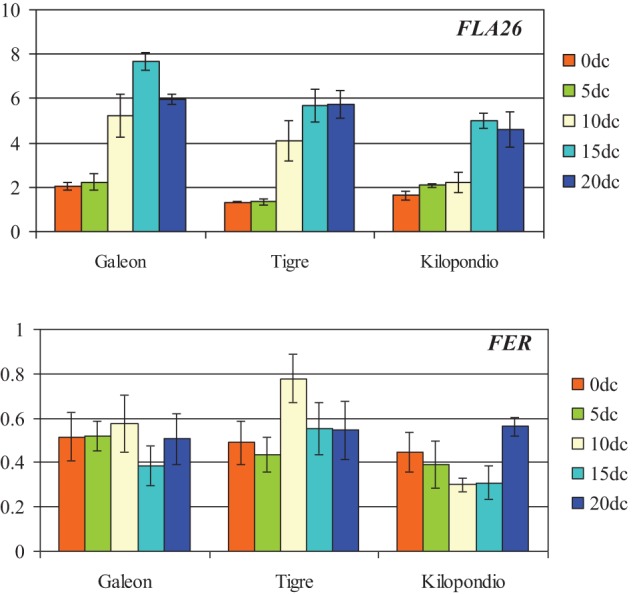
**Expression analysis of *FLA26* and *FERONIA* by qRT-PCR at 0, 5, 10, 15, and 20 days of culture (0dc, 5dc,10dc,15dc, and 20dc) in mature ovaries from bread wheat cultivars Galeón, Tigre, and Kilopondio**. The *y* axis indicates the gene mRNA level relative to Ta27771 used as control.

The expression patterns of *FLA26* and *FER* were also characterized by quantitative RT-PCR in mature ovaries from Galeón, Tigre, and Kilopondio cultivars that showed a similar inductive effect to Caramba. Analysis was performed at 0, 5, 10, 15, and 20 days of culture (0dC, 5dC, 10dC, 15dC, and 20dC) (Figure 7). The *FLA26* pattern of expression in the three genotypes was comparable to that observed in mature Caramba ovaries (Figure 6), as its expression increased during culture, reaching the highest level at 15dC in Galeón, and at 15dC–20dC in Kilopondio and Tigre. Expression levels of *FLA26* in Kilopondio and Tigre ovaries were the closest to those of Caramba, but Galeón showed the highest expression level at 15dC. In the analysis of *FER*, some similarities in the expression pattern of the three genotypes and Caramba were found as the level of expression at the beginning of the culture (0dC) was high. However, slight differences were also observed, since the level of expression remained close to the 0dC level, except at 15dC in Galeón and at 10–15dC in Kilopondio, with a decrease in their expression level, and a high increase at 10dC in Tigre.

## Discussion

### Ovary pre-conditioned and co-culture medium (OVPCM) increases wheat anther culture response

Our study shows that the utilization of an ovary pre-conditioned medium and ovary co-culture (OVPCM) has a positive effect on anther culture of high and medium-low responding cultivars of bread wheat, leading to a 6- and 11-fold increase in the numbers of embryos and green plants, respectively. Although it is well-known that ovary co-culture and/or an ovary pre-conditioned medium can be essential in wheat isolated microspore culture (for review see Zheng, 2003), few studies have addressed their effect on wheat anther culture response, even though this system is widely used for the routine production of doubled haploids (Jauhar et al., 2009; Lantos et al., 2013; Broughton, personal communication). Only Broughton (2008) conducted a comparative study of the use of ovary co-culture or ovary pre-conditioned medium, concluding that co-culture without pre-conditioning produced the highest number of green plants. Despite these data, we adopted the use of OVPCM as “nurse factors” that are immediately available at the time of anther culture, which is a great advantage. Furthermore, 5 days of ovary co-culture, which is the time used for ovary pre-conditioned medium in this study, was sufficient to produce enough “nurse factors” to induce microspore embryogenesis in isolated wheat microspore culture (Puolimatka and Pauk, 1999). Moreover, when isolated microspore culture was assayed in recalcitrant cultivars, ovary co-culture was not effective but an ovary pre-conditioned medium was essential (Zheng et al., 2002).

In this study, the effect of OVPCM depended on the genotype, having a greater effect on the medium-low responding cultivar Caramba than in the high responding cultivar Pavon. Thus, OVPCM raised the number of green plants up to 16 times and the percentage of regeneration 4 times in Caramba. These data are in accordance with those reported in microspores culture, showing a greater improvement in recalcitrant genotypes (Hu and Kasha, 1997; Zheng et al., 2002). Conversely, the study by Broughton (2008) in anther culture showed no interaction between the effect of ovary co-culture and anther genotype.

Previously, not much attention has been paid to the effect of the ovary genotype on isolated microspore culture in wheat, as the initial studies concluded that a wide range of genotypes were effective, and no clear differences between genotypes were shown (Bruins et al., 1996; Liu et al., 2002; Zheng et al., 2002). Thus, the ovaries from the same spikes from which the anthers are excised have traditionally been used for co-culture or OVCM. Surprisingly, in this study, a significant effect of the ovary genotype on microspore embryogenesis efficiency was observed. Caramba ovaries greatly increased the number of embryos and green plants and the percentage of green plants as compared to Pavon ovaries. As far as we know, only two references have previously suggested ovary genotype differences in the inductive effect on isolated microspore culture. Firstly, Mezja et al. (1993) mentioned the low capacity of Pavon ovaries in microspore induction, and secondly, Bruins et al. (1996) indicated that ovaries of one cultivar produced higher rates of microspore viability as compared to a mixture of ovaries from four cultivars.

It is well-known that microspores should be at a mid-late uninucleate stage to induce microspore embryogenesis efficiently (Datta, 2005). For ovary co-culture and pre-conditioned medium, immature ovaries from the same flowers that are used for anther culture have traditionally been used (Mezja et al., 1993; Hu and Kasha, 1997; Puolimatka and Pauk, 1999; Broughton, 2008). However, Zheng et al. (2002) indicated that ovaries from flowers containing early uninucleate to binucleate microspores were all effective in promoting microspore embryogenesis. In this study we have demonstrated that the ovaries' inductive effect depends on the developmental stage. Co-culture with mature ovaries enhanced the number of embryos and green plants by 25–46% respectively as compared to immature ovaries in both cultivars of wheat. The percentage of green plants was also significantly higher with mature ovaries. These results differ from those reported by Lu et al. (2008) showing that barley microspores co-cultured with florets containing microspores at uninucleate stage produced a higher number of embryos than those co-cultured with florets at binucleate stage. In our study, the effect of the ovary developmental stage depended on the ovary genotype. Immature ovaries from Pavon produced half as many green plants as mature Pavon ovaries, whereas mature ovaries from Caramba rendered only a slightly higher number of green plants than immature ovaries. Overall, the highest number of green plants was obtained with mature Caramba ovaries.

Taking into account that anther culture efficiency depends on the ovary genotype, it would be desirable to identify a donor ovary genotype that could produce the maximum induction rate and that could be used universally for co-culture in wheat anther culture. Therefore, ovaries from three agronomically important cultivars in Spain were compared with Caramba ovaries for OVPCM, obtaining a similar number of embryo and green plants with all. Unexpectedly, Tigre ovaries induced the highest number of DH plants, due to a high percentage of spontaneous chromosome doubling. It has been suggested that spontaneous doubling takes place by a nuclear fusion mechanism during stress treatment and culture in barley (Kasha et al., 2001; González-Melendi et al., 2005). Thus, “nurse factors” from Tigre ovaries seem to promote nuclear fusion during culture. The rates of spontaneous doubling have been described to be affected by the genotype, the type of stress treatment and the stage of the microspores at the time of culture (for review see Castillo et al., 2009). To our knowledge, this is the first time that the ovary genotype used for OVPCM has been reported to greatly affect chromosome doubling, however more studies are needed to clarify the mechanism through which Tigre ovaries lead to an increased nuclear fusion.

### Identification of candidate genes associated to the ovary inductive effect in OVPCM on microspore embryogenesis

Since early studies showed that the use of “nurse cultures” enhanced differentiation and proliferation in several systems (see review Boutilier et al., 2005), attempts have been made to identify these “nurse factors.” The nature of these compounds, when using ovary co-culture and/or ovary preconditioned medium systems, has been discussed extensively although few candidates have been recognized until now (Borderies et al., 2004; Asif et al., 2014). The differences in microspore embryogenesis efficiency due to ovary genotype and developmental stage found in this study were decisive in identifying the molecular mechanisms associated with the inductive effect of the ovaries. Five genes were selected as candidate genes among those connected with ovary biological functions, ovary metabolism during the co-culture, microspore embryogenesis or cell wall modification.

Three of the candidate genes, *TAA1b*, *FLA26*, and *WALI6* have been associated with wheat microspore embryogenesis response (Sánchez-Díaz et al., 2013). *TAA1b* was induced in the first stages of microspore embryogenesis when most of the microspores presented a star-like morphology, whereas *FLA26* and *WALI6* were induced at later stages, in multicellular structures confined inside the exine wall. However, *TAA1b*, that encoded a fatty acyl-CoA reductase involved in signaling (Wang et al., 2002), and *WALI6*, a cysteine-rich serine protease inhibitor induced by wounding or metal stress (Richards et al., 1994), showed expression patterns that could not be associated to the inductive effect of the ovaries. In this sense, *TAA1b* was only strongly induced in Pavon and *WALI6* in mature ovaries of both genotypes. Another gene that exhibits an expression pattern that was not associated with the inductive effect was *CGL1*. This gene is involved in the biosynthesis of complex glycans that are part of membrane glycoproteins and secretory proteins (von Schaewen et al., 2008) and showed the highest level of expression in immature Pavon ovaries, which had the lowest inductive effect. Finally, the expression of *FLA26* and *FER* showed a pattern characterized by having a higher level in Caramba than in Pavon ovaries at the onset of culture. In addition, the expression levels of both genes increased during culture, reaching a maximum at 15 days and being higher in Caramba mature ovaries, which showed the highest induction rate. Therefore, this pattern could be associated to the inductive effect of the ovaries. Nevertheless, some differences should be highlighted as *FER* expression levels were almost 10 times lower than *FLA26*, and *FER* expression was maintained or increased slightly during culture whereas *FLA26* showed a higher level at 15–20dC.

The period at which the “nurse factors” should be present in the medium has been widely studied (Hu and Kasha, 1997; Puolimatka and Pauk, 1999; Zheng et al., 2002). It has been established that the effect of the ovaries begins between 5 and 10 days of co-culture and that this initial effect is related to the rates of dividing microspores or maintenance of embryogenic development. However, the number, size and embryos frequency increase after 21 days of co-culture (Hu and Kasha, 1997). Thus, the beneficial effect of ovary factors extends throughout the induction culture period, probably due to the presence of different “nurse factors” with different functions (Hu and Kasha, 1997) that meet the requirements of the induced microspores at each stage of the embryonic development. In this sense, the induction of a different set of functional genes throughout wheat microspore embryogenesis has been described recently (Sánchez-Díaz et al., 2013).

The receptor-like kinase FERONIA (FER), which belongs to the CrRLK1L-1 subfamily, was originally identified for its role as a communication factor during fertilization acting in termination of pollen tube growth (Huck et al., 2003; Rotman et al., 2003; Escobar-Restrepo et al., 2007). Therefore, its function would fit with the idea that ovary factors are related to ovary biological functions as suggested by Mezja et al. (1993). However, other receptor-like kinase, ERECTA, associated with cell-cell signaling related to cell proliferation and growth in ovaries, anthers and embryos (Shpak, 2013) was only expressed in fresh ovaries at 0 days of culture (data not shown).

In recent years, new data suggest that FER plays a more general role in complex processes, intervening as a master regulator of cell growth (Yu et al., 2012, 2014; Duan et al., 2014). These data are consistent with the fact that the “nurse factors” were not ovary-specific (Puolimatka and Pauk, 1999). FER could act in signal transduction as a co-receptor to enhance the activity of other receptor kinase, or as receptor of the peptide secreted RALF (rapid alkalinization factor) (Haruta et al., 2014; Kessler et al., 2015). It has been reported that FER participates in a feedback signaling network in growing cells but also in stress conditions, through an interplay with the brassinosteroid signaling (Guo et al., 2009; Hofte, 2015). In this network, the cell wall secretions promote cell expansion, leading to the activation of mechanoreceptors that trigger an increase in cytosolic Ca^2+^, alkalinization of the apoplast, ROS production and growth inhibition (Rojas et al., 2011). All components of this signaling network are important to microspore embryogenesis induction. Increased Ca^2+^ concentration resulted in a higher osmolality and a larger number of embryo-like structures and plant production (Hoekstra et al., 1997). A shift toward alkalinization upon embryogenesis induction could be behind the cytoskeletal rearrangements (Pauls et al., 2006). Żur et al. (2014) suggested that a certain H_2_O_2_ threshold was important for successful microspore embryogenesis induction, and ROS-induced signal transduction has been associated with the stress treatment (Jacquard et al., 2006; Żur et al., 2009). Finally, the incorporation of brassinosteroids in the culture medium of *Brassica* microspores significantly increased the percentage of embryogenesis (Ferrie et al., 2005).

Compounds secreted by the cell wall are also involved in microspore embryogenesis. In fact, these compounds have been among the first to be associated with the induction effect of ovary co-culture and/or ovary preconditioned medium (Borderies et al., 2004; Matthys-Rochon, 2005). Among them, AGPs, a large family of cell surface hydroxyproline-rich glycoproteins (HRGPs), were found to induce wheat microspore embryogenesis (Letarte et al., 2006). *FLA26* encoded for a fasciclin-like arabinogalactan protein (FLAs), a subclass of AGPs that contains an AGP-like glycosylated domain and two fasciclin-like domains (Faik et al., 2006). The FLAs are widely distributed in various plants but little information is available as compared to other AGP families. Nevertheless, FLAs are known to be involved in cell-to-cell communication associated with plant growth and development (Johnson et al., 2003; Shi et al., 2003; Faik et al., 2006; Zang et al., 2015). The *FLA26* gene was highly expressed in microspore embryogenesis, fresh anthers, excised embryo and roots, indicating a general function in wheat (Sánchez-Díaz et al., 2013). However, other *FLAs* genes (*FLA25* and *FLA14*) that were also expressed in microspore embryogenesis induction, were not expressed in ovaries in culture (data not shown). Also, it is known that different AGPs are expressed in reproductive tissues (Cheung et al., 1995; Coimbra et al., 2008). Accordingly, in this study the two *AGPs* analyzed *(AGP5* and *AGP9)* were expressed in fresh ovaries but not during culture (data not shown).

In this study we have demonstrated that the use of ovary pre-conditioned medium and ovary co-culture increased the efficiency of green doubled haploid plant production in bread wheat anther culture. In addition, ovary genotype and stage of development are critical factors, increasing embryogenesis induction and chromosome doubling percentages at early stages of microspore embryogenesis, and enhancing green plant regeneration in later stages. A fasciclin-like arabinogalactan protein, FLA26, and the ovary signaling gene *FERONIA* have been associated with the inductive effect of the ovaries. Our results represent a breakthrough in identifying the molecular mechanisms associated with microspore embryogenesis induction, and suggest new approaches to increase the efficiency of anther culture that could have special relevance in bread wheat plant breeding.

### Conflict of interest statement

The authors declare that the research was conducted in the absence of any commercial or financial relationships that could be construed as a potential conflict of interest.
